# Waterless Dyeing of Polyamide 6.6

**DOI:** 10.3390/polym16111472

**Published:** 2024-05-22

**Authors:** Semiha Eren, İrem Özyurt

**Affiliations:** 1 Textile Engineering Department, Bursa Uludag University, Bursa 16059, Türkiye; 2 Karesi Polyester Company, R&D Center, Bursa 16370, Türkiye; iremozyurt@rbkaresi.com.tr

**Keywords:** polyamide (PA), supercritical carbon dioxide (scCO_2_), dyeing

## Abstract

Waterless dyeing of polyamide 6.6 using scCO_2_ (supercritical carbon dioxide) was investigated. PA (polyamide) fibers can be dyed with various dyes, including disperse dyes. The conventional aqueous dyeing process uses large amounts of water and produces polluted water. Considering these environmental issues, waterless dyeing of fibers is a forefront issue, and utilization of supercritical carbon dioxide (scCO_2_) is a commercially viable technology for waterless dyeing. This study tested PA6.6 (polyamide 6.6) dyeing in scCO_2_ at 100 °C 220 bar pressure for 45 min. Color measurements and color fastness tests were performed, as well as tensile strength, scanning electron microscope (SEM) analysis, and Fourier transform infrared spectroscopy (FTIR) analysis. PA6.6 fabrics yielded higher K/S (color strength, the Kubelka–Munk equation) values with larger molecular weight dye and almost the same color strength with medium and small-sized dyes, demonstrating the ability of dyeing in a supercritical environment without water as a more environmentally friendly dyeing option compared to conventional dyeing.

## 1. Introduction

Polyamide (PA), a polymer held together with amide bonds (-NH-CO-), also known as nylon, represents an essential type of synthetic fibers. Although various types of PA are produced, PA6 (polyamide 6, (-HN-(CH_2_)_5_-CO)_n_) and PA6.6 (polyamide 6.6, (-HN-(CH_2_)_6_-NHCO-(CH_2_)_4_-CO-)_n_) are the most essential types of PA fibers [1]. Aramids are also an important type of PA fibers, built totally (or at least 85%) from link-adjacent aromatic monomers [2].

The production of synthetic fibers increased to 80.4 million tons in 2022, much higher than the 7.3 million tons of cellulosic fibers [3]. Meanwhile, the annual production of polyamide fibers worldwide in 2022 was 6.21 million tons [4].

Synthetic fibers play a pivotal role in the textile industry due to their versatility and utility across various applications, whereas natural fibers like cotton fall short due to inherent limitations. Synthetic fabrics offer several advantages, including enhanced strength, elasticity, reduced wrinkling, lightweight characteristics, and rapid drying capabilities. These attributes make synthetic fabrics well suited for sportswear and other performance-oriented applications [5].

Polyester, the most widely utilized synthetic fiber, has distinctive properties attributed to its aliphatic and aromatic molecular components and regular molecular structure. These fibers have low moisture absorbency, high resiliency, dimensional stability, excellent wear, weather, light resistance, good abrasion resistance, and effective blending capabilities with cotton. The primary polyester for fiber production is Poly(ethylene terephthalate) (PET), favored not only for its end-use properties and cost-effectiveness but also for its ease of physical and chemical modification, which can enhance positive attributes while suppressing negative ones. Moreover, polyester finds application in composite materials due to its advantages, such as low cost, dimensional stability, and low cure temperature. These attributes enable its use in simplified molds, contributing to its role in composite material development alongside other thermoset polymer matrices [6,7]. Polypropylene (PP) is an olefin polymer known for its low melting point and excellent insulation properties. PP fibers are widely used in various applications such as rope manufacturing, concrete reinforcement, medical sutures and meshes, hygiene products, home furnishings, automotive interiors, and surgical disposables. They are valued for their strength, durability, biocompatibility, and cost-effectiveness, making them versatile and practical across different industries [8,9,10]. Polyethylene (PE) stands out as a fundamental polymer in various industries due to its low density and high tensile strength. These properties make PE reinforcements highly appealing for numerous applications. Commercially available ultrahigh-molecular-weight polyethylene fibers are extensively used in various commercial and defense applications, showcasing their versatility and effectiveness in modern industries [11,12]. Acrylic fibers are derived from polymers with a minimum of 85% acrylonitrile content in their monomers. These polymers contain highly polar nitrile groups along the polyacrylonitrile chain, allowing for strong intermolecular interactions. Acrylic fibers serve as the primary precursor for carbon fiber production. Carbon fibers (CFs) have seen extensive use in composite materials across industries like aviation, space, automotive, sports equipment, energetics, medicine, and construction. PAN-based CFs are produced through the stabilization and carbonization of specialized PAN fibers [13,14,15,16].

PA textiles exhibit exceptional mechanical properties, making them highly versatile for various applications, from everyday apparel to technical textile uses [17]. Polyamide fibers are of significant commercial importance in the textile industry and are widely used in producing yarns, garments, industrial textiles, and carpets [18]. PA stands out due to its lightweight, softness, durability, and moderate sweat absorption characteristics. These properties enable the production of knitted fabrics suitable for lingerie, sportswear, and socks. In addition to producing fabrics that are lightweight, breathable, and resistant to wear, PA is also used to produce carpets, upholstery, and other household items [19,20]. PA is also used for different applications; meta-aramid, para-aramid, and polyamide-imide were used to create nanofibrous membranes [21]. Multifunctional electrospun membranes of PA6 loaded with silica (SiO_2_) and titanium dioxide (TiO_2_) nanoparticles were tested for water purification [22]. Bioactive polyamide fibers, obtaining PA6 fibers modified with acetanilide and copper ions, can be utilized for medicine and environmental protection to produce filters [23]. Adding sodium polyacrylate and recycled PA fibers into cement improved heat storage properties and energy-efficient insulation [24].

The mechanical properties of PA fibers in the long term decrease due to chemical aging, and they can undergo hydrolysis when in contact with aqueous media for a long time [25]. On the other hand, PA can be mechanically or chemically recycled through thermal depolymerization, which makes it a sustainable material [26].

The dyeing characteristics of PA are influenced by amino end-group content and physical structure. Both amine and carboxylic acid terminal groups are present in PA. Hence, anionic and cationic dyes may be used for dyeing. PA can also be dyed with disperse dyes. The dyeing of PA with acid dyes is similar to the dyeing of wool. During dyeing by acid dyes, amino groups of the molecular chain interact with the sulfonic acid group of the dye. Since the molecular chains of PA have no side chains, the maximum number of end groups is two per chain, having a limited number of sites for absorption of acid dyes, resulting in difficulty in obtaining dark shades. In regular PA, half of the chain ends are amino groups, and the other half are carboxyl groups; this can be changed by terminating the polymerization reaction, and deeper dyeing by acid dyes can be achieved if most chain ends are amine groups. Direct dye application on PA is similar to that of acid dyes, but they do not offer significant advantages over acid dyes. Chrome dyes yield heavy shades with good wet fastness properties. However, color matching may be complex due to shade changes after metallization. Metal-complex dyes also have high saturation values, good fastness washing, and light properties. Cationic dyeable PA can also be achieved by incorporating acidic groups in the polymer backbone. These modifications help the dyer produce tone-on-tone and multicolor patterns in a single dyebath using more than one dyeability type PA fibers, primarily for carpet manufacturing [27,28]. The dyeing of PA with natural dyes is also reported in the literature. Microwave was used to isolate natural colorants from saffron and safflower for dyeing PA [29], and rhubarb flower parts were also used to produce a brown hue on PA6 fabric [19].

Disperse dyeing of PA in water is usually performed at pH 5–6.5 at the boil in the presence of a leveling agent. However, it is restricted to pale and medium shades due to limited fastness properties. The variations in end groups of PA do not affect disperse dyeing since disperse dyes are held by physical bonding forces. The application method is simple, and dyeing is level. However, disperse dyeing of PA in heavy depths yields moderate light fastness and low washing fastness. Good leveling disperse dyes with low molecular weight are widely used for dyeing nylon hoses, tights, carpets, and woven and knitted fabrics [28,29]. The conventional aqueous dyeing process uses large amounts of water and produces polluted water containing dyestuff and auxiliaries after dyeing. Moreover, microplastic pollution is a serious global issue, and PA is one of the microplastic types that has been determined [30,31]. Considering these environmental issues, waterless dyeing of fibers is at the forefront. Utilization of supercritical carbon dioxide (scCO_2_) is a commercially viable technology for waterless dyeing. Substituting water with supercritical carbon dioxide (scCO_2_) can preserve energy, lower water use, and prevent pollution in textile dyeing [32]. Dyeing of various fibers in supercritical carbon dioxide (scCO_2_) was reported for polyester [33,34,35,36], wool [37], cotton [38,39], and cellulose acetate [40], but it is currently commercially available for polyester. Dyeing of PA in supercritical carbon dioxide (scCO_2_) was also reported [41,42]. Elmaaty et al. (2015) [41] applied a series of disperse azo dyes with potential antibacterial properties to dye the PA6 fabric using supercritical carbon dioxide (scCO_2_) as the medium. Five azo disperse dyes were used; the studied pressures were 50–100 and 150 bar pressures, and temperatures were 80–100 and 120 °C. The highest K/S was reached at 150 bar and 120 °C compared to other studied circumstances. Penthala et al. (2022) [42] designed and synthesized a reactive disperse dye, which featured a tricyanopyrrolidone moiety coupled with a triazine reactive group to dye PA6.6 at 250 bar and 120 °C in supercritical carbon dioxide (scCO_2_). The dye exhibited good values of K/S (12.17%) and reflectance percentage (92%) on the dyed PA6.6 fabrics, attributed to the formation of a covalent bond between the reactive group of the dye molecule and the -NH group present in PA6.6 fibers.

In this study, dyeing of PA6.6 in supercritical carbon dioxide (scCO_2_) was investigated using three commercially available disperse dyes of low, medium, and high energy level on two types of PA6.6 fabrics to observe the effect of dye’s molecular weight on dyeing of PA6.6 fabrics in supercritical carbon dioxide (scCO_2_).

## 2. Materials and Methods

### 2.1. Materials

The study used two plain woven fabric constructions containing 100% PA6.6 yarns woven at Karesi Polyester Company (Bursa, Turkiye). For the dyeing process, the three types of disperse dyes were used. The properties of the fabric samples and disperse dyes are given in Table 1 and Table 2, respectively.

**Figure 1:**
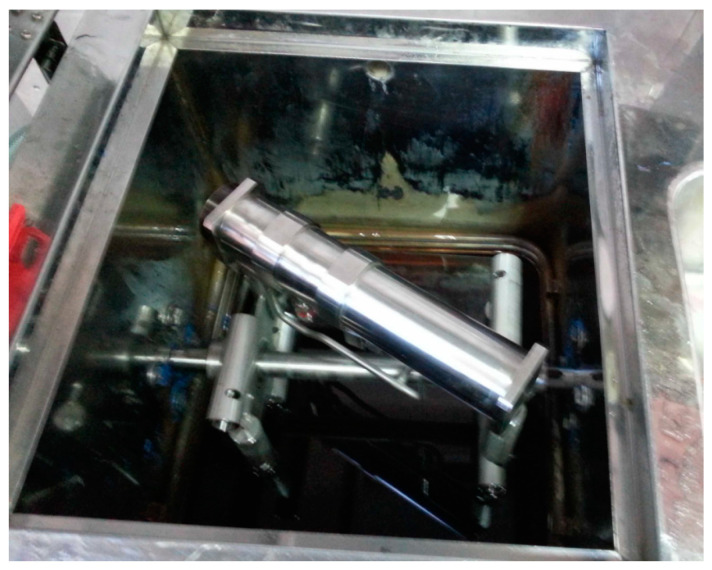
The 290 mL volume laboratory-type supercritical carbon dioxide (scCO_2_) processing vessel placed inside the oil heating apparatus.

PA6.6 woven fabric samples were dyed in a waterless environment using a 290 mL volume laboratory-type supercritical carbon dioxide (scCO_2_) processing vessel (DyeCoo Lab-dye, Weesp, The Netherlands). It is crucial to fill the tube with liquid CO_2_ to be used as a solvent for the successful dyeing of the fabric. Cooling is necessary to ensure the tubes are filled with CO_2_. The cooling process is required for the liquid carbon dioxide to be filled into the tube safely after placing the samples and the dyes into the tube. A Vestel SD 200 (Vestel, Istanbul, Turkiye) deep freezer was used for the tube cooling. To maintain the temperature of the dyeing process, the DyeCoo scCO_2_ processing tube is placed inside a Rapid Xiamen bath (Rapid Xiamen Model H-12, Xiamen, China) containing polyethylene glycol (PEG), as shown in Figure 1.

### 2.2. Methods

#### 2.2.1. Conventional Dyeing

The samples were dyed using 2% (omf-over the mass of fabric) dye. In the aqueous dyeing process, 0.2 g/L soda ash, 2 g/L leveling agents, and acetic acid (pH 6) were used alongside the dye substance. The dyeing process took place at 100 °C for 45 min. After dyeing, washing was carried out using 2 g/L nonionic detergent at 70 °C for 20 min, followed by rinsing with water at 70 °C for 10 min.

#### 2.2.2. Dyeing in scCO_2_ Media

The samples were dyed using 2% (omf-over the mass of fabric) dye. Dyeing was performed at 100 °C 220 bar pressure for 45 min. The experiments involving constant density ranges were conducted based on the guidelines provided by the DyeCoo scCO_2_ application information graph and technical information sheet, which outline the relationship between pressure, temperature values, and corresponding tables. The dyeing procedures in a supercritical carbon dioxide (scCO_2_) medium followed the outlined steps. Initially, fabric samples were enveloped around a beam and inserted into steel tubes with a volume of 290 mL, engineered to endure pressures up to 30 MPa and temperatures up to 140 °C. They underwent precooling in a deep freezer (−18 °C) for approximately 15 min to facilitate carbon dioxide infusion into the prepared tubes. The precise amount of necessary carbon dioxide was computed based on temperature and pressure data from the National Institute of Standards and Technology (NIST) Chemistry WebBook and introduced into the precooled tubes. Finally, the filled tubes were introduced into an oil bath dyeing apparatus to commence the dyeing process. The density range of 550–600 kg/m^3^ was selected in this study, which is the range advised for polyester by DyeCoo [33,49,50].

#### 2.2.3. Tests and Analysis Performed

##### Color Measurement

After dyeing, the color strength of the samples was determined by calculating the K/S values based on the reflectance values at the maximum absorbance (λmax) wavelength using a Konica Minolta (Tokyo, Japan) spectrophotometer. The computation of K/S values are based on the Kubelka–Munk equation, *K*/*S* = (1 − *R*)^2^/2*R*, where R represents the reflectance of the dyed fabric sample at the absorption maximum (λmax), while K and S denote the absorption and scattering coefficients, respectively. The measurements were conducted under illuminant D65 with a 10° standard observer.

##### COD (Chemical Oxygen Demand) Test

The chemical oxygen demand (COD) assessment of process waste after conventional dyeing was measured according to the standard colorimetric method (Standard Methods 5220 D: Closed reflux, colorimetric method). The wastewater obtained post-conventional dyeing was added to each COD test tube. Subsequently, the prepared COD tubes were incubated in a thermostatic reactor at 148 °C for 2 h. Following removal from the thermostatic reactor, COD values in mg/L were quantified using a UV–visible spectrophotometer (Merck; Spectroquant Pharo 300, Darmstadt, Germany).

##### Washing Fastness

Washing fastness was determined according to the ISO 105:C06 A2S [51] test method. The samples were cut to dimensions of 100 × 40 mm and subsequently stitched onto a multifiber test fabric of the same dimensions. A washing solution was prepared using ECE detergent according to the standard method. Each test sample and stitched multifiber test fabrics, steel balls, and the prepared washing solution were placed inside steel tubes. These tubes were inserted into the washing fastness test device. After the washing cycle, the samples were dried and subjected to evaluation using a Konica Minolta (Japan) spectrophotometer. The assessment was based on a scale from 1 (lowest) to 5 (highest)

##### Perspiration Fastness

Perspiration fastness was determined according to the ISO 105-E04 [52] test method. Acidic and basic solutions were prepared and added to containers for each sample. The samples’ multifiber portion was submerged at the bottom of the solution and allowed to soak for 30 min. Then, samples were retrieved and sandwiched between two acrylic plates to place in an empty perspirometer. A standard weight was applied to the perspirometer. The assembled perspirometer was subsequently positioned upright within an incubator set to a temperature range of 37 ± 2 °C and left for four hours. Upon completion of the incubation period, the samples were removed, dried, and subjected to evaluation using a Konica Minolta (Japan) spectrophotometer. The assessment was based on a scale from 1 (lowest) to 5 (highest).

##### Tensile Strength

Tensile strength tests were performed according to ISO 13934-1 [53]. The fabric specimens were prepared with the dimensions of the test. Each fabric sample was securely clamped between a fixed and movable jaw and then subjected to tension until rupture occurred. The resultant data were quantified in strength units, measured explicitly in newtons (N).

##### Scanning Electron Microscope (SEM) Analysis

All sample surface images were examined using the Hitachi TM330Plus (Hitachi, Tokyo, Japan) model SEM device.

##### Fourier Transform Infrared Spectroscopy (FTIR) Analysis

The samples’ infrared (IR) spectra were obtained using the Shimadzu IRTracer-100 (Shimadzu, Kyoto, Japan) model FT-IR spectroscopy method.

## 3. Results

### 3.1. Color Values

**Figure 2:**
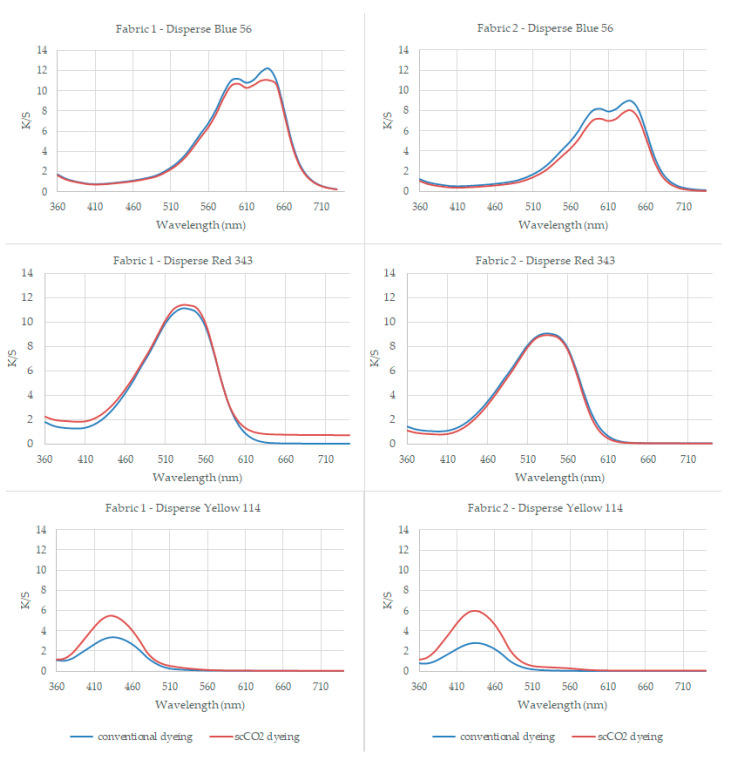
The plot of K/S values for the dyed samples across the visible spectrum range.

The K/S values of the samples at their maximum reflectance wavelengths (640 nm for blue, 530 nm for red, and 430 nm for yellow color) are presented in Table 3. The K/S plotting of the dyed samples among visible range is given in Figure 2.

Table 3 presents the results of dyeing PA6.6 fabric with Disperse Blue 56, Disperse Red 343, and Disperse Yellow 114 at 100 °C for 45 min. The maximum color uptake, expressed as K/S values, was observed at specific wavelengths for different fabrics.

The K/S values at the absorption maximum (λmax) for conventionally aqueous dyed samples using the low-energy dye Disperse Blue 56 were observed to be 0.15 higher for fabric 1 and 0.93 higher for fabric 2 when compared to the K/S values of the scCO_2_ dyed samples.

The K/S values at the absorption maximum (λmax) of samples utilizing the medium-energy dye Disperse Red 343 exhibited minor differences compared to the results obtained with the low-energy Disperse Blue 56 dye. Specifically, for conventionally aqueous dyed samples, the K/S value was 0.14 higher for fabric 2 compared to scCO_2_ dyed samples. Conversely, for scCO_2_ dyed samples, the K/S value was 0.59 higher for fabric 1 compared to conventionally aqueous dyed samples.

Contrary to the previous observations with low and medium-energy dyes, the high-energy Disperse Yellow 114 dye led to a notable shift in results. Specifically, at the absorption maximum (λmax), the K/S values for scCO_2_ dyed samples were significantly higher, with a difference of 2.15 for fabric 1 and 3.19 for fabric 2 compared to conventionally aqueous dyed samples.

The K/S values for the dyed samples were plotted across the visible spectrum range and are presented in Figure 2 to visualize the variation in K/S values corresponding to changes in the molecular size (energy-level) of the disperse dyes utilized. Upon evaluation, it was observed that PA6.6 fabrics dyed with Disperse Yellow 114 in a waterless environment achieved better dyeing results compared to conventionally dyed samples, resulting in a significantly darker appearance. The dyeing process took place at 100 °C for 45 min for both aqueous and scCO_2_ dyeing, but the color yield for high-energy dye (CI Disperse Yellow 114) was higher. The 220 bar pressure applied during scCO_2_ dyeing helped the large high-energy level disperse dye diffuse into the PA fiber.

Results showed that the fabric absorbed low-, medium-, and high-energy dye substances, with low-molecular-weight dye substances being absorbed more effectively. When Figure 2 is examined, the K/S plotting of the conventionally aqueous dyed samples mostly overlapped by scCO_2_ dyed samples among visible wavelength range for CI Disperse Blue 56 (low-energy-level disperse dye) and CI Disperse Red 343 (medium-energy-level disperse dye). However, contrary to this, the K/S plotting of the conventionally aqueous and scCO_2_ dyed samples differed among the visible wavelength range for CI Disperse Yellow 114 (high-energy-level disperse dye).

When Figure 2 is examined in detail, blue plotting (the K/S values of the conventionally aqueous dyed samples) was slightly higher for low-energy-level (smaller dye molecule) disperse dye (CI Disperse Blue 56) compared to the orange plotting (the K/S values of the scCO_2_ dyed samples); it came to an equilibrium for the medium-energy dye (CI Disperse Red 343) but the orange plotting (the K/S values of the scCO_2_ dyed samples) became higher than blue plotting (the K/S values of the conventionally aqueous dyed samples) for the high-level (largest dye molecule) disperse dye (CI Disperse Yellow 114). The orange plotting on the K/S curves of CI Disperse Yellow 114 (high-energy-level disperse dye) was higher than that of conventionally aqueous dyed samples, especially around the maximum reflectance wavelength of 430 nm, indicating that more dyes were fixed to the PA samples during scCO_2_ dyeing.

The results clearly demonstrate that larger disperse dyes with a high-energy level exhibit improved dye uptake under scCO_2_ conditions. These results are consistent with findings from previously published research on dyeing polyester and polypropylene using scCO_2_. Özcan and Özcan (2005) [54] studied polyester dyeing under scCO_2_ and reported that the solubility of dye increases in direct proportion to rising pressure, attributed to decreased intermolecular distances. This reduction enhances solvent–solute interactions, thereby boosting solubility. Consequently, dissolved dye molecules readily diffuse into the amorphous regions of swollen fibers, leading to their adsorption onto the fibers. This mechanism ultimately results in a significant adsorption of dye onto the fabric. [54].

Elmaaty et al. (2019) [55] investigated the dyeing of polypropylene fabrics under scCO_2_ and reported that the color strength (K/S value) of dyed polypropylene fabrics exhibited a gradual increase with rising system pressure. Theoretically, at lower pressures, the density of scCO_2_ fluid is low, which could lead to improved swelling of polypropylene fibers as pressure increases. This enhanced swelling promotes the penetration and diffusion of dyes into the amorphous regions of the fibers, consequently contributing to increased color strength [55].

### 3.2. Washing Fastness Test Results

**Figure 3:**
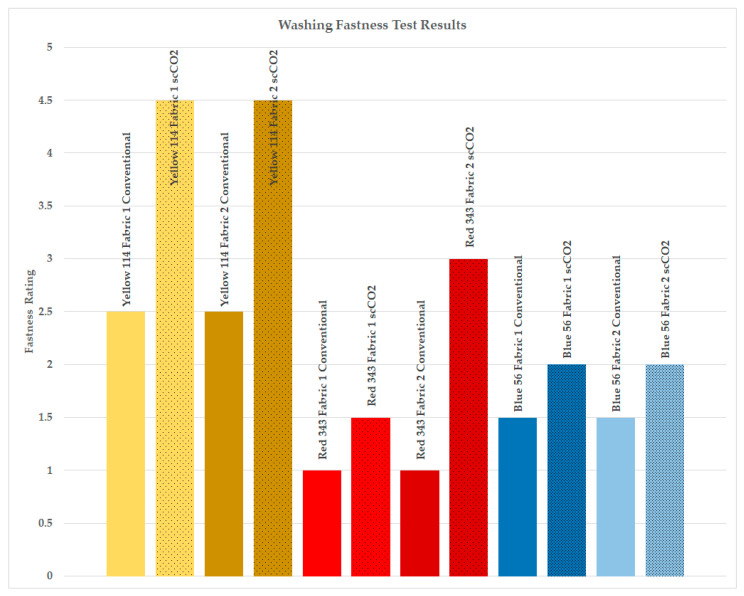
Washing fastness test results, staining ratings on nylon strip of the adjacent multifiber test fabric.

Washing fastness test results for the dyed samples are presented in Figure 3.

Fastness properties are evaluated by assessing staining on an adjacent white sample concurrent with the fading of the colored specimen being tested. The degree of staining is measured using a gray scale, which comprises nine pairs of nonglossy grey and white strips. These strips represent varying levels of perceived staining, providing corresponding fastness ratings ranging from 5 (excellent) to 1 (poor). This method is commonly used to gauge the amount of staining on adjacent undyed fabrics during fastness tests. A standardized fabric known as multifiber is frequently used as the adjacent fabric in color fastness tests to evaluate material color transfer. Multifiber comprises yarns from various generic fiber types, each forming a strip at least 15 mm wide to ensure consistent fabric thickness. The individual strips in multifiber include cellulose diacetate, bleached cotton, polyamide, polyester, acrylic, and wool, making it a versatile and widely used material for assessing color fastness properties.

As seen in Figure 3, the washing fastness ratings of PA6.6 fabrics dyed with a waterless scCO_2_ process increased compared to samples dyed using conventional methods. The variations in staining were particularly notable on acetate, nylon, and polyester fibers of the multifiber test fabric, which aligns with expectations for dyeing with disperse dyes. Notably, the highest degree of staining was observed on the nylon fibers of the multifiber test fabric. Consequently, Figure 3 exclusively presents the staining observed on the nylon fibers of the multifiber test fabric, providing a clearer representation of the staining outcomes.

The washing fastness ratings on nylon increased to an average of 2.92 for scCO_2_ dyed samples from 1.67 for conventionally aqueous dyed samples. The average fastness rating for all fibers was 3.75 for scCO_2_ dyed samples, whereas it was 3.07 for conventionally aqueous dyed samples, indicating an increase of 0.68 in gray scale degree for scCO_2_ dyed samples.

The average improvement in fastness ratings for scCO_2_ dyed samples, specifically using high-molecular-weight disperse dye (Disperse Yellow 114), was 2 degrees higher compared to conventionally aqueous dyed samples. For medium-energy dye (Disperse Red 343), the average increase was 1.25 degrees, while for low-energy level disperse dye (Disperse Blue 56) with smaller dye molecules, the average improvement was 0.5 degrees. In line with the findings illustrated in Figure 2, which demonstrate that larger disperse dyes with higher energy levels exhibit enhanced dye uptake under scCO_2_ conditions, the washing fastness ratings for these larger disperse dyes showed a more pronounced increase as a result of dyeing in supercritical carbon dioxide (scCO_2_).

These results are in agreement with the literature. Liao et al. [56] stated that the lightfastness and washfastness of PA6.6 fabrics dyed using their synthesized disperse-reactive yellow dyestuff with supercritical carbon dioxide were excellent. Schmidt et al. [57] reported that the fastness properties of dyed PA were comparable to polyester.

### 3.3. Perspiration Fastness Test Results

**Figure 4:**
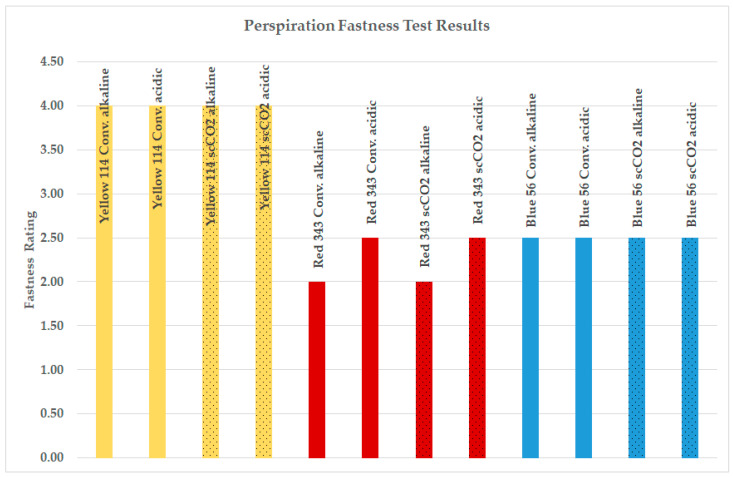
Perspiration fastness test results for fabric 1.

The perspiration fastness test results for the dyed samples of fabric 1 are displayed in Figure 4. As the results for fabric 2 were identical to those of fabric 1, only the results for fabric 1 are presented, for easier comprehension. The perspiration fastness test assesses the color resistance of dyed textiles against acidic and alkaline perspiration. This test simulates conditions where garments come into contact with heavy perspiration, potentially leading to localized discoloration. A colored textile specimen, alongside other fiber materials to test for color transfer, is wetted with simulated acid perspiration solution (pH 5.5) and alkaline perspiration solution (pH 8.0) and subjected to mechanical pressure before drying slowly at an elevated temperature. After conditioning, the fiber materials are assessed for any color transfer.

As seen from Figure 4, the perspiration fastness data of PA6.6 fabrics dyed with the waterless scCO_2_ method were as successful as samples dyed using conventional methods. Polyamide (PA) is distinguished by its lightweight nature, soft texture, durability, and moderate sweat absorption properties. These attributes make it ideal for manufacturing knitted fabrics for lingerie, sportswear, and socks. Therefore, the perspiration test is commercially demanded for these fabrics. The mean perspiration rating for conventional dyeing was 3.80, whereas for scCO_2_ dyeing, it was slightly lower at 3.78. The highest perspiration fastness test ratings were observed for the high-energy-level Disperse Yellow 114 dye. The findings depicted in Figure 4 illustrate that supercritical carbon dioxide (scCO_2_) dyeing produces comparable perspiration fastness ratings when compared to traditional aqueous dyeing methods.

### 3.4. Chemical Oxygen Demand (COD) Values of the Aqueous Dyeing Bath

The objective of this study was to achieve successful dyeing of PA6.6 fibers using disperse dyes without the use of water. Chemical oxygen demand (COD) indicates the level of organic pollutants in contaminated water.

Dyeing under supercritical carbon dioxide (scCO_2_) eliminates the need for water, thereby avoiding associated water pollution. However, evaluating contamination levels linked to conventional aqueous dyeing offers a more informative assessment of the water pollution prevented by incorporating scCO_2_ dyeing. By comparing these contamination levels between the two methods, we gain a more comprehensive understanding of their respective environmental impacts. Therefore, chemical oxygen demand (COD) analyses were conducted on the dyebath effluents of conventionally aqueous dyed PA6.6 samples.

Dyebath effluent from the conventional dyeing process was analyzed using COD analysis. The average COD load of the dyebath effluents for both fabrics and all dyes was 5235 mg/L. No wastewater is generated in dyeing processes conducted in a waterless environment. Therefore, it can be concluded that approximately 5235 mg/L of COD loads can be prevented from being in the environment by scCO_2_ dyeing.

### 3.5. Tensile Strength Test Results

Demonstrating that the proposed scCO_2_ dyeing method does not harm PA fabric is crucial, especially for its use in garments. Conducting strength tests, SEM, and FTIR analyses helps ensure that there are no significant differences between scCO_2_ dyeing and conventional aqueous dyeing in this respect. Choosing conventionally aqueous-dyed samples as the reference is justified by their widespread commercial use, while scCO_2_ dyeing represents an innovative and investigative approach.

**Figure 5:**
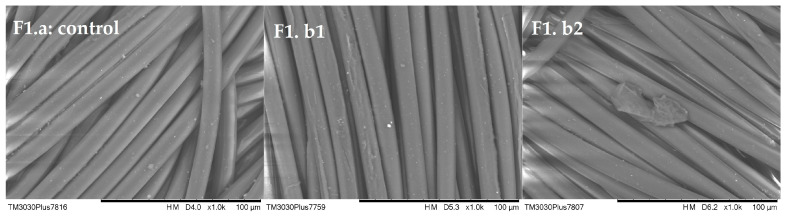
SEM images of the samples of fabric 1 (**F1.a**) undyed fabric (control), (**F1.b1**) conventional Disperse Yellow 114, and (**F1.b2**) scCO_2_ Disperse Yellow 114.

The results of the tensile strength tests revealed a slight decrease in fabric strength under scCO_2_ dyeing conditions, with an average of 0.63 kN observed for both fabric 1 and fabric 2 dyed using three disperse dyes. In contrast, conventional aqueous dyeing yielded an average strength of 0.85 kN for the same fabrics and dyes. However, this minor reduction in tensile strength is unlikely to impede the practical use of the fabric. These findings are corroborated by the SEM images presented in Figure 5, which depict undamaged fabrics despite the observed strength difference.

### 3.6. Scanning Electron Microscope (SEM) Analysis Results

SEM images of the undyed, conventionally aqueous dyed, and scCO_2_ dyed samples are presented in Figure 5.

Microscopy studies were undertaken to establish whether or not the process conditions of scCO_2_ dyeing damaged the PA6.6 fibers. No apparent splitting or fibrillation was observed on the surface of either the fibers in fabric 1 or the fibers in fabric 2 when the fibers after dyeing were compared with the control fibers. There was indeed no fiber damage, and no difference existed between samples. This shows that dyeing conditions using the scCO_2_ method are suitable for dyeing PA6.6 fiber and do not damage this fiber.

The SEM images depicted in Figure 5 correspond to the tensile strength test results, which demonstrate the absence of any detrimental effects following dyeing in supercritical carbon dioxide (scCO_2_) under a pressure of 220 bar.

### 3.7. FTIR Analysis Results

**Figure 6:**
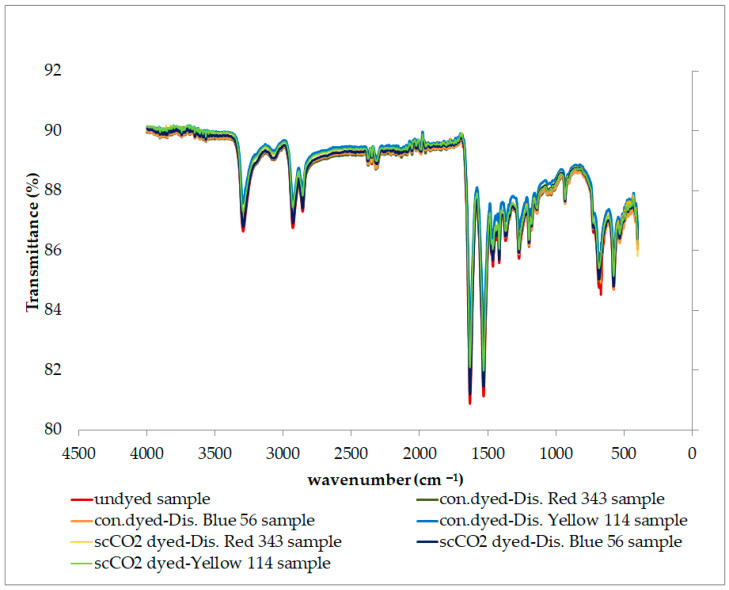
FTIR plotting of the fabric 1 samples.

The structural characterization of different PA6.6 samples dyed with varying dyes in conventional aqueous and scCO_2_ environments was carried out by analyzing functional groups using Fourier transform infrared spectroscopy (FTIR). FTIR plotting of the undyed, conventionally aqueous dyed, and scCO_2_ dyed samples of fabric 1 are presented in Figure 6; the results were similar for fabric 2. The peaks observed in the graphs in Figure 6 are evidence of the characteristic peaks of PA6.6. The band at 3300 cm^−^^1^ is attributed to the hydrogen-bonded N─H stretching [58,59,60,61,62,63,64,65]. The 2933 and 2862 cm^−^^1^ peaks are associated with methylene groups’ stretching or stretching vibration (CH_2_). The appearance of absorption peaks at 1656 cm^−^^1^ and 1540 cm^−^^1^ belongs to the –N-H stretching band of the amine group. These peaks correspond to the characteristic absorption peaks (stretching vibration of the carbonyl group) and the amid II bonds (bending vibration of the amino group) of PA6.6. [57,58,59,60,61]. The peaks between 1370 and 1200 cm^−^^1^, combined with the C─N stretching and N─H deformation vibrations, show the amid III region associated with the N─H in-plane bending. The peaks at 1169 and 959 cm^−^^1^ correspond to the skeletal motion of CO─NH and the in-plane vibration of CO─NH, respectively [61,66]. When Figure 6 is examined, it is observed that the type of dyeing process (aqueous or scCO_2_ environment) did not have a considerable effect that would disrupt the structure of PA 66.

The strength tests, scanning electron microscopy (SEM), and Fourier transform infrared spectroscopy (FTIR) analyses confirm that there are no significant differences between scCO_2_ dyeing and conventional aqueous dyeing in terms of fiber damage. This validates the safety and feasibility of incorporating scCO_2_ dyeing for PA6.6 without compromising fiber integrity.

### 3.8. Limitations of the Study

A limitation of this study could be related to the fact that all dyeing was carried out in a waterless environment using a 290 mL volume laboratory-type supercritical carbon dioxide (scCO_2_) processing vessel (DyeCoo Lab-dye, Weesp, The Netherlands), and other types of supercritical carbon dioxide (scCO_2_) processing apparatus were also present [41,42]. The most important limitation is the small size of the sample. Since the experiments were conducted at the laboratory, uneven dyeing could not always be observed in small samples, but evenness is an essential issue for textile dyeing. Therefore, an industrial scale testing of the dyeing PA6.6 in supercritical carbon dioxide (scCO_2_) may be helpful.

## 4. Conclusions

This study investigated and compared the dyeing of PA6.6 with three commercial disperse dyes of low, medium, and high energy level in the scCO_2_ dyeing system without water with the conventional aqueous dyeing process. In the supercritical environment, waterless dyeing is crucial for preventing fiber damage and achieving successful dyeing, requiring the right combination of temperature and time. This study demonstrates that PA6.6 can be dyed successfully without using any water.

In the literature reviewed, two studies explored the dyeing of PA (polyamide) using supercritical carbon dioxide (scCO_2_). Elmaaty et al. (2015) investigated the application of a series of disperse azo dyes with potential antibacterial properties on PA6 fabric using scCO_2_ as the medium. They utilized five distinct azo disperse dyes across various pressures (50–100 and 150 bar) and temperatures (80–100 and 120 °C), noting the highest color strength (K/S) at 150 bar and 120 °C compared to other experimental conditions. Penthala et al. (2022) developed and synthesized a reactive disperse dye incorporating a tricyanopyrrolidone moiety coupled with a triazine reactive group specifically for dyeing PA6.6 fabric using scCO_2_. This dyeing process occurred at 250 bar and 120 °C, resulting in dyed PA6.6 fabrics with favorable color strength (K/S of 12.17%) and reflectance percentage (92%). These outcomes were attributed to the formation of a covalent bond between the reactive group of the dye molecule and the -NH group present in PA6.6 fibers. In this study, three commercial disperse dyes with varying energy levels were employed to evaluate the scCO_2_ dyeing performance of PA 6.6. Additionally, selected tests were crucial for the commercialization of the process.

The results showed that the fabric absorbed low-, medium-, and high-energy dyes, with low-molecular-weight dye substances absorbed more effectively. The K/S values of the conventionally aqueous dyed samples were by scCO_2_ dyed samples for CI Disperse Blue 56 (low-energy-level disperse dye) and CI Disperse Red 343 (medium-energy-level disperse dye). However, contrary to this, the K/S value of the conventionally aqueous and scCO_2_ dyed samples differed for CI Disperse Yellow 114 (high-energy-level disperse dye). The scCO_2_ dyed high-energy disperse dye (CI Disperse Yellow 114) yielded higher color values than the aqueous dyed sample.

Test and analysis results did not show detrimental damage of PA6.6 after scCO_2_ dyeing. The colorfastness results for the scCO_2_ dyed samples were outstanding. As a measure of water pollution and environmental impact, the COD values exhibited 5000–6000 mg/L values for the dyebath effluent of conventional aqueous dyeing, exhibiting another advantage of scCO_2_ dyeing, which does not use water or create effluent. The successful dyeing of PA6.6 fabrics without using any water in scCO_2_ environments emerges as a promising and forward-looking solution.

It is crucial to demonstrate that the proposed scCO_2_ dyeing method does not have any detrimental effects on PA fabric, especially for its use in garments. Conducting strength tests, SEM, and FTIR analyses helps ensure that there are no significant differences between scCO_2_ dyeing and conventional aqueous dyeing. Choosing conventionally aqueous-dyed samples as the reference is justified by their widespread commercial use, while scCO_2_ dyeing represents an innovative and investigative approach.

The utilization of supercritical carbon dioxide (scCO_2_) for dyeing is an emerging technology, primarily employed for disperse dyeing of polyester. This study represents a step towards the commercial dyeing of polyamide (PA) fibers using scCO_2_, contributing to expanding the scope of this technology. Additionally, the color measurement results from the study demonstrated higher color values for scCO_2_ dyeing compared to conventional aqueous dyeing methods. This finding can guide the selection of the appropriate dyeing method, particularly for large-scale dyeing operations.

## Figures and Tables

**Table 1:** Physical properties of samples.

Specimen	Warp Yarn (tex)	Weft Yarn (tex)	Warp Density (cm/warp)	Weft Density (cm/weft)	Weight (g/m^2^)
Fabric 1	16.66	16.66	30	24.5	115
Fabric 2	4.44	8.88	57	36	70

**Table 2:** Properties of disperse dyes used in the study [43,44,45,46,47,48].

Disperse Dyes	Chromophore Group	Energy Level/Molecular Weight (g/mol)	Chemical Structure
CI Disperse Blue 56	Anthraquinone	Low/349	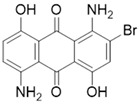
CI Disperse Red 343	Azo	Medium/410	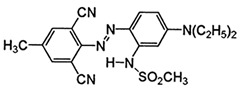
Disperse Yellow 114	Azo	High/424	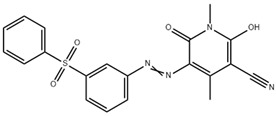

**Table 3:** The K/S values at their maximum reflectance wavelengths of the samples.

Dyestuff	Dyeing Method	Maximum Absorption Wavelength	Fabric 1	Fabric 2
Disperse Blue 56	Conventional	K/S 640 nm	12.20	8.98
	scCO_2_		11.05	8.05
Disperse Red 343	Conventional	K/S 530 nm	11.12	9.07
	scCO_2_		11.71	8.93
Disperse Yellow 114	Conventional	K/S 430 nm	3.34	2.78
	scCO_2_		5.49	5.97

## Data Availability

Data are contained within the article.
